# Spindle lesions in the thyroid: a cytological and histological review

**DOI:** 10.1007/s00428-025-04095-5

**Published:** 2025-04-11

**Authors:** Angela Feraco, Federica Vegni, Belen Padial Urtueta, Qianqian Zhang, Elena Navarra, Antonino Mule, Liron Pantanowitz, Esther Diana Rossi

**Affiliations:** 1 Division of Anatomic Pathology and Histology-Fondazione, Policlinico Universitario “Agostino Gemelli”-IRCCS, Università Cattolica del Sacro Cuore, Largo Francesco Vito, 1–00168 Rome, Italy; 2 Department of Pathology, Pittsburgh University, Pittsburgh, PA USA

**Keywords:** Fine needle aspiration cytology, Spindle cell lesions, Squamous cell carcinoma, Thyroid malignancies, Immunocytochemistry, Personalized medicine

## Abstract

The majority of thyroid lesions are of epithelial origin that exhibit a typical follicular and/or papillary growth pattern. The occurrence of a predominantly spindle cell lesion is uncommon in the thyroid gland and is likely to be misdiagnosed in cytological or histological samples, which may impact patient management. The diagnosis is made by finding a significant amount of spindle cells, which may be combined in some cases with other morphologic features. It is important to recognize if these spindle cells have benign or malignant features. The differential diagnosis for such lesions includes mesenchymal neoplasms (e.g., solitary fibrous tumor) and non-mesenchymal tumors (e.g., anaplastic thyroid carcinoma). The morphologic interpretation of such lesions can be problematic due to their rarity, pathologists’ limited experience, overlapping cytomorphologic features, and challenges selecting and interpreting appropriate ancillary studies. This review discusses most of the thyroid entities showing spindle cell features, emphasizing their cytological and histological findings of relevance to the recent Bethesda system for reporting thyroid cytopathology and WHO classification of endocrine tumors.

## Introduction

Thyroid lesions can be classified as primary epithelial, primary non-epithelial or metastatic origin. The vast majority of these lesions are of epithelial origin, including benign and malignant entities. Most thyroid lesions are characterized by a papillary and/or follicular growth architecture. Only a minority show unusual growth patterns [1, 2], such as a predominant solid or spindle cell component, as well as a peculiar biphasic pattern (Table 1).

**Table 1:** Distribution of a spindle pattern in thyroid lesions

Pattern	Benign	UMP	Malignant
Minimal spindle pattern	FA	HTT	PTC
Extensive spindle pattern	SFT, SMT, schwannoma, FDCT	HTT	PTC, ATC, SMT, MPNST, FDCT, CMTC, MTC, angiosarcoma, Fibrosarcoma, SS, US, CEFTE, SETTLE
Complete spindle pattern	SFT, SMT, schwannoma		ATC, angiosarcoma, SMT, MPNST, CMTC, MTC, SS, US, CEFTE, SETTLE

*FA* follicular adenoma, *HTT* hyalinizing trabecular tumor, *PTC* papillary thyroid carcinoma, *SFT* solitar fibrous tumor, *SMT* smooth muscle tumor, *FDCT* follicular dendritic cell tumor, *ATC* anaplastic thyroid carcinoma, *MTC* medullary thyoid carcinoma, *MPNST* malignant peripheral nerve sheath tumors, *CMTC* cribriform-morular thyroid carcinoma, *SS* synovial sarcoma, *US* undifferentiated sarcoma, *CEFTE* carcinoma of the thyroid with Ewing family tumor elements, *SETTLE* spindle epithelial tumor with thymus-like differentiation

The recent WHO classification of endocrine tumors, published in 2022, has divided thyroid tumors into new categories that better define their cellular origin and pathological features. Of note, a major change in the new WHO classification is categorizing primary squamous cell carcinoma of the thyroid as a subtype of anaplastic carcinoma [3]. Among the follicular-derived epithelial lesions, several may have a significant spindle cell component which might be problematic toward rendering an accurate cytological and histological interpretation. Both benign and malignant epithelial and mesenchymal lesions can present with a significant spindle cell component. To correctly diagnose a spindle cell lesion of the thyroid, ancillary techniques may likely be needed including immunohistochemistry (IHC), immunocytochemistry (ICC), and molecular testing.

The differential diagnoses of spindle lesions of the thyroid are broad and include spindle follicular adenoma, follicular neoplasms with spindle cell metaplasia, papillary thyroid carcinoma (PTC) with spindle cell metaplasia, PTC with fibromatosis/fasciitis-like stroma, cribriform morular thyroid carcinoma, medullary thyroid carcinoma (MTC), undifferentiated carcinoma, spindle epithelial tumor with thymus-like elements (SETTLE), as well as various benign and malignant mesenchymal tumors.

This review discusses the cytological and histological interpretation of diverse spindle cell lesions that may be encountered in the thyroid gland. The role of ancillary studies will be reviewed as part of the diagnostic algorithm for such lesions.

### Solitary fibrous tumor (SFT)

SFT was first described as a pleural tumor by Klepper and Rabin in 1931 [4–10]. Since then, it has been found in several organs, including the liver, parotid gland, and rarely thyroid gland. The first description by Taccagni et al. of this mesenchymal lesion involving the thyroid gland included three cases that manifested with a nodular goiter. Since then, around 48 such cases of this rare entity have been documented [6–15].

Patients who have a SFT of their thyroid typically range in age between 28 and 68 years, without any specific gender association. Their clinical history frequently documents a long-standing goiter that steadily grows despite L-thyroxine therapy [9]. Large nodules, sometimes measuring more than 30 mm in diameter, may cause dysphagia. Unlike pleural and certain extrapleural SFTs, which can cause fasting hypoglycaemia due to insulin-like growth factor production, patients with a thyroid SFT have not shown signs or symptoms of low glucose plasma levels. Physical examination reveals a firm and enlarged thyroid. The ultrasound (US) appearance is that of a large, hypoechogenic, and solid nodule.

**Fig. 1:**
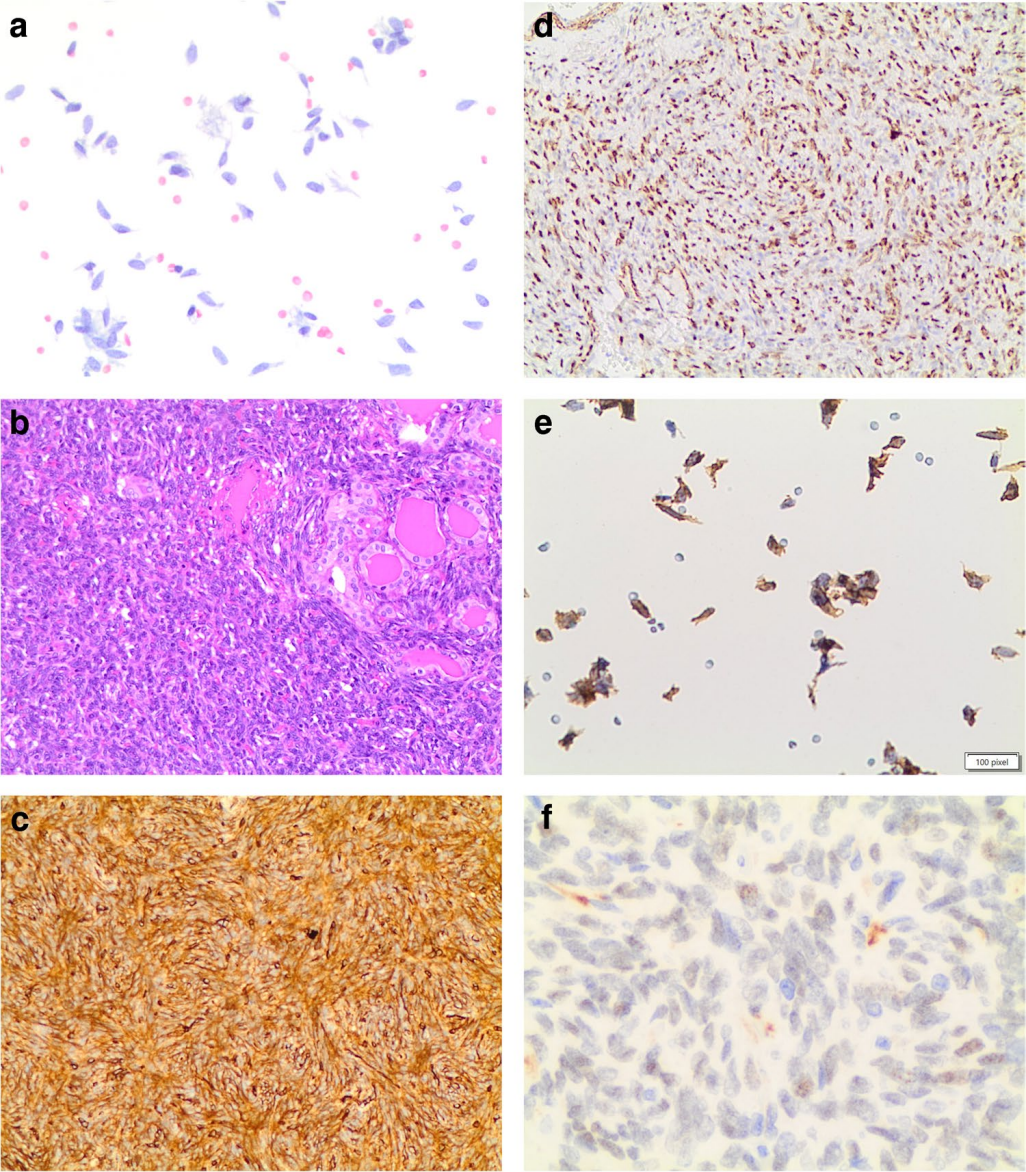
**a, b, c, d**: panel **a** shows the cytological features of a solitary fibrous tumor in the thyroid (PAP stain 40 ×). The cells are dischoesive and fusiform. Panel **b** shows the histological deatils of the same SFT (H&E 40 ×). The panels **c** and **d** show the positivity for CD34 (membranous and cytoplasmic) and STAT6 (nuclear) on histology, while panel **e** shows CD34 positivity on liquid-based-stored material (**a** and **b** 40 ×)

Fine-needle aspiration (FNA) is often non-diagnostic due to the presence of only a few dispersed spindle cells and minimal colloid [6, 13], leading to a difficult interpretation (Fig. 1a). The gross pathology features of thyroid SFTs often show a large, well-defined, solid tumor that is white to gray in color and seldom tan [9, 11]. Histologically, these neoplasms are characterized by solid growth of spindle-shaped, fibroblast-like cells with a haphazard distribution (“patternless”). Neoplastic cells can permeate the thyroid parenchyma in small follicle-dissecting fascicles or in nodules [7, 8, 11]. The spindle cells have eosinophilic cytoplasm and contain variously shaped nuclei such as round, oval, elongated, and spindle-shaped, with finely distributed chromatin and inconspicuous nucleoli (Fig. 1b). Tumor cellularity varies. Typically, they have a mitotic rate of < 4/10 high-power fields. There is often no necrosis or vascular invasion [7–15, 17]. Demicco et al. defined a new risk stratification scheme for SFT based on the combined evaluation of patient age, tumor size, and mitotic activity to predict risk of metastasis [18].

SFT cells show positive staining for bcl- 2, CD34, STAT6 (Fig. 1c–f), and, in rare cases, actin. SFTs in the thyroid gland do not react with epithelial markers such keratins, thyroglobulin, thyroid transcription factor- 1 (TTF- 1), epithelial membrane antigen (EMA), as well as neuron-specific markers (e.g., S- 100 protein, neurofilaments, and acetylcholinesterase), neuroendocrine markers (calcitonin, neuron-specific enolase, chromogranin, synaptophysin, and tyrosine hydroxylase), nor muscle cell markers (e.g., smooth muscle myosin, desmin, and basal lamina components).

Applying the Demicco criteria, all known cases of SFT reported in the thyroid, except for one case, belonged to the low-risk group of patients, and all of them were successfully treated with surgical excision alone [17, 18].

### Post-fine-needle aspiration spindle cell nodule of the thyroid (PSCNT)

For the correct identification of this entity, the patient’s clinical history of a prior FNA targeting their thyroid gland is imperative. PSCNT is a reactive spindle cell lesion**,** showing a nodular architecture, discovered in patients who have previously undergone an FNA of a thyroid nodule [18–24]. Various iatrogenic spindle cell proliferations in extrathyroidal body regions may arise after surgical treatment, including nodular fasciitis, proliferative fasciitis, inflammatory pseudotumor, and postoperative spindle cell nodules [18–23]. The identification of a spindle nodular and/or pseudonodular reaction to surgery was described in 1984 by Proppe et al. [26], who reported eight individuals that developed spindle cell nodules in the genitourinary tract following surgery.

In 1999, Baloch and colleagues reported ten patients with PSCNT, including seven women and three males aged 49 to 74 years [25]. A spindle cell proliferation was observed inside both benign and malignant thyroid nodules after a 2-week to 2-month gap between biopsy and/or surgery. Lesions attributed to PSCNT varied in size from 3 to 10 mm (mean 5.6 mm; median 5.5 mm). In the paper by Baloch et al., PSCNTs were not encapsulated and primarily found in the middle of pre-existing thyroid nodules. Light microscopy showed that these lesions consisted of plump spindle cells with elongated nuclei, vesicular chromatin, inconspicuous nucleoli, and ill-defined cytoplasmic boundaries. In addition, tiny thin-walled capillaries and a mixed inflammatory infiltrate with histiocytes, lymphocytes, and hemosiderin-laden macrophages were observed. The degree of cellularity ranged from mild to exuberant, with irregular collagen deposition and focal hyalinization. This variability may be attributed to the timing of the FNA and subsequent surgery, the size of the biopsy needle, and an unknown host response.

To specify, this type of spindle nodular/pseudonodular component represents part of a nodule undergone previous FNAC, or in few cases a complete spindle FNAC reactive substituition of the nodule. For its diagnosis, the previous history of an FNA is crucial.

By immunohistochemistry, the spindle cell component reacted to smooth muscle actin, but not to cytokeratin. CD68, a histiocytic marker, marked the macrophage component in all cases. Immunohistochemical features thus confirm the spindle myofibroblastic origin [26].

PSCNTs are harmless. Nonetheless, they may be misdiagnosed since morphologically they might resemble sarcoma due to their high cellularity, plump spindle cells, and enlarged nuclei. However, they show a low mitotic rate. Their proper diagnosis can be made based on the relative circumscription of the lesion, lack of cell pleomorphism, FNA biopsy history, and immunohistochemistry results.

## Follicular lesions with spindle component

Follicular thyroid lesions with spindle cells, whether predominant or simply localized, are extremely rare and might provide significant diagnostic challenges [27]. Few studies have discussed the entity of multinodular goiters with spindle features. Typically, these lesions occur in patients showing a median age of 63 y/o, with an equal gender distribution and an average size of nodules at around 2.6 cm.

These cases are distinguished by the presence of several benign thyroid follicular nodules of varying size, as well as a spindle cell component with elongated or plump nuclei arranged primarily in fascicles, which can also exhibit pleomorphism [29–31]. The spindle follicular cells are primarily located at the periphery of these nodules. There is no inflammatory or fibrous reaction, and mitotic activity and necrosis are inconspicuous. However, because of the biphasic morphology of these lesions, a panel of immunohistochemical markers (including Ki- 67) should always be performed to avoid misdiagnosing them as a malignant lesion [32].

**Fig. 2:**
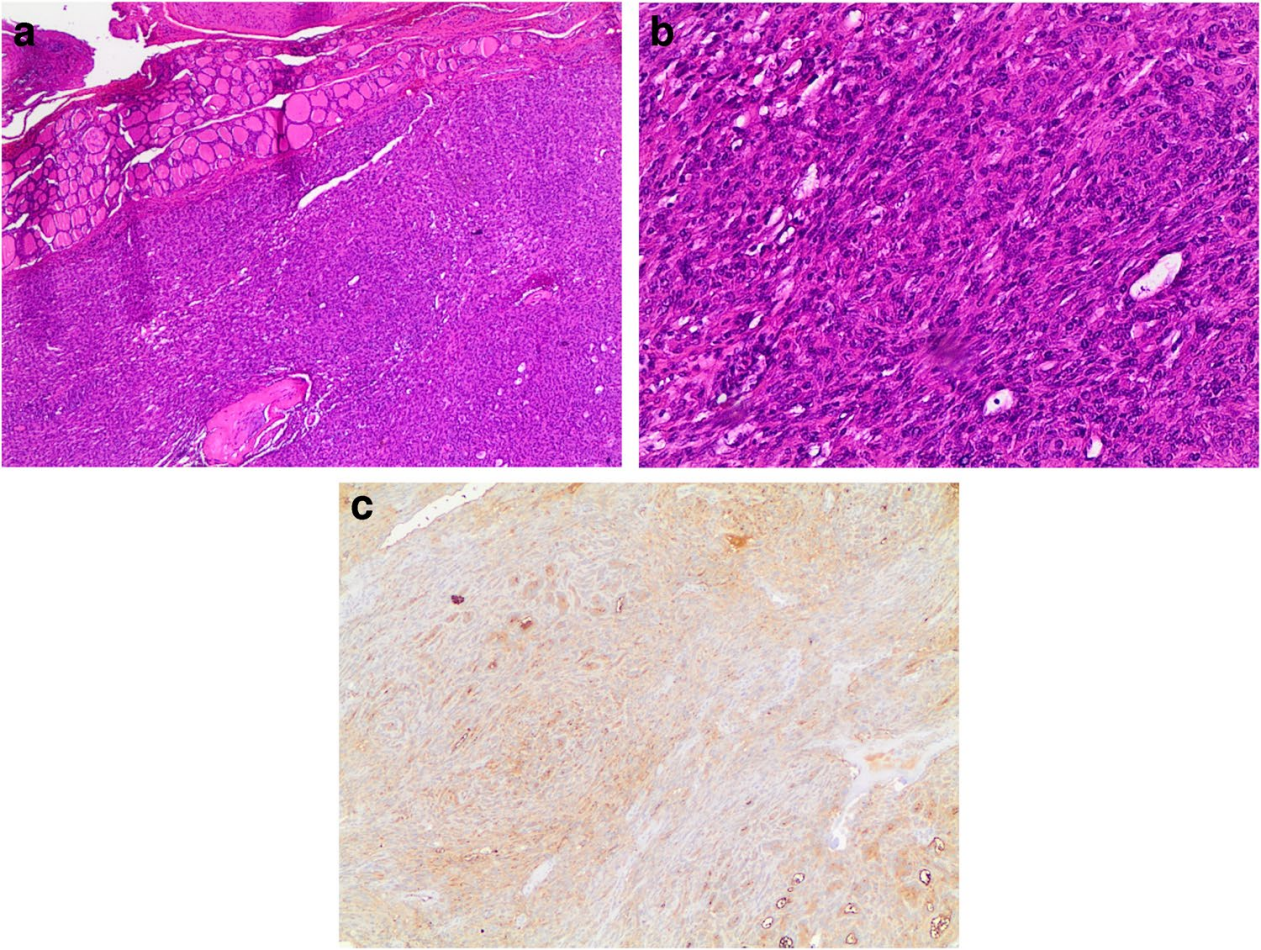
**a** and **b:** the panels **a** and **b** show the details, at different magnification, of spindle cells in an adenoma of the thyroid. No evidence of capsular and vascular invasion (H&E 20 ×, and 40 ×). Panel **c** shows the positivity for thyroglobuline (40 × **a** and **b**)

The differential diagnosis includes thyroid follicular adenoma and follicular carcinoma with spindle cells. Both of these follicular lesions share a microfollicular cellular architecture with cuboidal cells delimiting the follicles. Follicular adenoma is a relatively frequent thyroid lesion that is commonly associated with euthyroidism in patients [33]. Previously, ten examples of follicular adenomas with spindle cell features were documented in the literature. These unusual lesions, defined by a microfollicular architecture and lack of capsular or vascular invasion, contained a spindle cell component that ranged from 1 to 95% of the nodule (Fig. 2a–b). The spindle-shaped cells were detected mostly near the nodule’s perimeter or intermingled within the microfollicular component, as well as within focal areas of solid growth. Hyalinization of the stroma and vascular walls was frequently observed. Notably, no considerable mitotic activity or necrosis was observed [29–31].

Magro et al. have also described thyroid follicular adenoma with meningioma-like characteristics, in which the encapsulated tumour had bland spindle cells grouped in whorls around blood vessels [28]. These traits, which are similar to a transitional meningioma, likely represent a type of spindle cell metaplasia that may occur in benign and malignant thyroid tumors [28]. The average age of patients manifesting with this type of nodule is 57 years, with a female preponderance of 7:3. These nodules exhibit no substantial mitotic activity [28]. Pericytic-like follicular adenoma is another variant characterized by an abundance of ovoid/spindle cells arranged in a pericytic fashion (i.e., cells wrapped around blood vessels), expanding the cytomorphological spectrum of follicular thyroid neoplasms [34, 35]. An immunohistochemistry panel including thyroglobulin (Fig. 2c), TTF- 1, PAX8, vimentin, cytokeratins (clone AE1/AE3), calcitonin, CD31, and CD34 with low MIB1/Ki67 will reflect the benign and metaplastic nature of these follicular lesions. According to Shikama et al., the close relationship between the spindle cell areas and the adenomatous regions shows that the spindle cells likely originated from the adenoma itself rather than de novo [36].

Data from the literature (between 1996 and 2022) indicates that there have been at least three cases of follicular thyroid carcinoma with spindle cell metaplasia reported. Apart from the typical features of malignancy, these lesions were characterized by a proliferation of spindle cells arranged in a haphazard pattern, often within more solid areas. These spindle cells were mixed with thyroid follicular cells that lack the nuclear characteristics of papillary thyroid carcinoma, as well as frequent atypia and mitotic figures. Given the rarity of spindle cell metaplasia in follicular thyroid carcinoma (FTC), it is critical to identify the follicular origin of these spindle components. In fact, the differential diagnosis can be broad, including both reactive and neoplastic entities. Hence, the correct diagnostic evaluation requires the use of immunohistochemistry [27]. Given the rarity of these entities and the limited understanding of the molecular mechanisms behind this change, the prognosis for the existence of a spindle component in these lesions remains uncertain [37].

In 2017, Arnoux et al. identified the novel entity “non-invasive follicular tumour of the thyroid with papillary-like nuclear characteristics” (NIFTP), further defined by Nikiforov et al. [38]. NIFTP lesions too have been seen to have spindle cell growth [39]. In such a case, the nodule showed all of the typical NIFTP features, but also had an intralesional nodule with a spindle cell characteristic. These spindle cells had the same immunoprofile as follicular cells (i.e., positivity to thyroglobulin, TTF- 1, HBME- 1, beta-catenin, and focal galectin- 3 staining), except for vimentin positivity and E-cadherin negativity, which were only found in the spindle cell portion, implying a common origin of the two components, and allowing the diagnosis of NIFTP with focal spindle cell metaplasia to be rendered [39].

Haroon Al Rasheed et al. also reported an example of NIFTP in which a spindle cell component was identified. The nodule in their patient was composed of two separate components: mostly packed with epithelioid cells showing nuclear features indicative of papillary thyroid cancer, and a minority of spindle cells mixed in among the epithelioid cells. In this case, no substantial mitotic activity nor necrosis was found. TTF- 1, PAX- 8, and HBME- 1 showed strong immunohistochemical positivity in both components. Positive thyroglobulin and CK7 only stained the spindle component. With these findings, a single origin for the two cell types was proposed, once again validating the diagnosis of NIFTP with spindle cell metaplasia [40].

Chen et al. described a case of a 58-year-old woman who had a 3-cm thyroid nodule previously diagnosed as “low-grade malignant neoplasm with features of follicular carcinoma, spindle cell variant,” which was then treated with radioiodine and hormonal therapy. The ultrasound examination of the neck in this patient revealed a 3.3-cm latero-cervical lymph node, of which a FNAC showed a population of many spindle cells with elongated and plump nuclei, thin and granular chromatin, and indistinct nucleoli organized in fascicles with positivity for pan-CK, PAX8, thyroglobulin, and TTF- 1 and negativity for CEAm and calcitonin. The conclusive diagnosis was accordingly in favor of a metastatsis to this lymph node of the FTC with spindle cell metaplasia [41].

### Hyalinizing trabecular tumor (HTT)

**Fig. 3:**
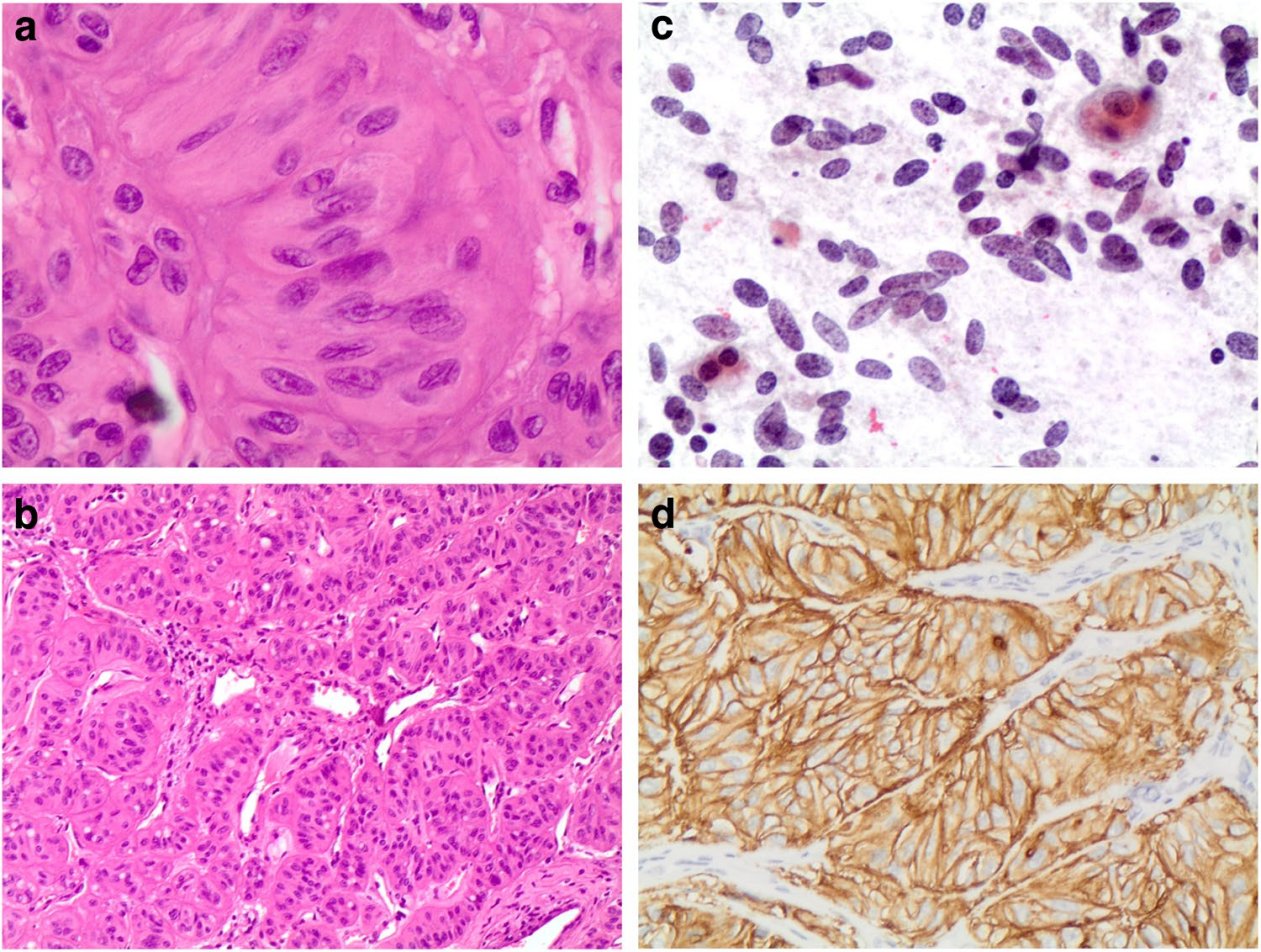
**a, b, c, d**: panels **a** and b show the morphological features of the hyalinizing trabecular tumor with the spindle appearance and the eosinophilic cytoplasma. Panel **c** shows the same case on a cytological smear with the typical spindle morphology. Panel **d** shows positivity for MIB1. (H&E 20 × AND 40 ×, Pap stain and **a** and **b**)

HTT is a rare follicular-derived encapsulated slow-growing neoplasm with a trabecular pattern of growth, marked intratrabecular hyalinization and occasionally calcified extracellular matrix, composed of elongated cells arranged around capillaries in a paraganglioma-like fashion (Fig. 3a–b). HTT occurs in people younger than 30 years and shows a marked female predilection [42].

HTT mimics medullary carcinoma, paraganglioma, as well as PTC. The clinical behavior of HTT is generally benign; although, a few cases of HTT with nodal metastases, capsular, and/or vascular invasion have been reported. For this reason, the term “hyalinizing trabecular tumor” rather than adenoma has been preferred in recent years [43–48]. Several authors have hypothesized that HTT and PTC are related and may share similar histological features (i.e., intranuclear cytoplasmic inclusions, nuclear grooves, nuclear overlapping, perinuclear clearing, and psammoma bodies) as well as molecular features (RET/PTC rearrangements) [43, 49, 50]. However, the histogenesis of HTT remains controversial.

Some authors have defined distinctive criteria to differentiate HTT from a PTC in cytological samples (Fig. 3c), which include the presence of vague, curved nuclear palisading; radiating arrangement surrounding hyaline material; elongated cells; cell processes; ill-defined cell border; faintly stained and filamentous cytoplasm; yellow bodies; hyaline material in the background; lack of papillary architecture; and sheet-like arrangement [51] (Fig. 3c).

From a histopathology perspective, HTT is composed of spindle-shaped cells with abundant hyaline, acidophilic to amphophilic cytoplasm and distinct cell borders, arranged in nests and trabeculae within a delicately hyalinized fibrovascular stroma. Scattered cytoplasmic yellow bodies are a common finding. Although most tumors are encapsulated, occasionally capsular invasion, infiltration into adjacent normal thyroidal tissue and even lymph node metastases have been reported. By immunohistochemistry, HTT cells are strongly positive for thyroglobulin, TTF- 1, MIB- 1 (Fig. 3d), and negative for calcitonin [52, 53]. Some HTTs are reported to stain for RET/PTC oncoprotein [49], CK19, HMW-CKs [54, 55], and galectin [56].

Nikiforova et al. demonstrated that there is a strong association between GLIS fusions and HTT. In their series of 14 cases, they found that GLIS rearrangements, particularly PAX8-GLIS3, are highly prevalent in HTT but not in PTC. Specifically, PAX8-GLIS3 was seen in 93% of HTTs.

This neoplasm behaves in a benign manner in almost all cases, and, therefore it initially was named trabecular hyalinizing adenoma.

### Smooth muscle tumors

Smooth muscle tumors (SMTs) include benign (leiomyoma) and malignant (leiomyosarcoma) neoplasms whose origin is controversial. The most possible origin is the development from smooth muscle cells mostly located within the intrathyroidal vessels [58–63]. Primary and secondary SMTs in the thyroid glands are quite rare, while metastases from leiomyosarcomas in other sites (e.g., leg, pulmonary artery, uterus), albeit rare, have been observed more frequently [58–70]. Nonetheless, it is challenging to recognize thyroid metastatic SMT from a primary SMT due to their clinical, pathology, and immunohistochemical similarities. In such cases, correlation with a previous clinical history of leiomyosarcoma in an extrathyroidal location is thus of paramount importance.

Data from the literature reported five cases of primary leiomyoma [58–62] and 11 cases of primary leiomyosarcoma [58–65] within the thyroid gland. Some authors suggest that primary thyroid SMT originates from smooth muscle-walled capillaries especially found at the periphery of the thyroid [63, 70]. Of note, Tulbah et al. [65] reported the case of a child with immunodeficiency presenting with an Epstein-Barr virus-associated leiomyosarcoma involving the thyroid.

Leiomyoma typically affects younger patients compared to leiomyosarcoma, which develops in the sixth and seventh decades of life. Benign thyroid SMTs are more common in females (female/male ratio, 4:1), likely attributed to the production of estrogen receptor proteins by these tumors [70]. On the other hand, malignant SMTs do not exhibit a gender preference. However, both tumors present as an indolent mass often in one thyroid lobe, developing either slowly or rapidly. As expected, patients with leiomyosarcoma typically experience pressure symptoms, particularly at the tracheal level due to the infiltrative pattern of these neoplasms [64]. These lesions have been accompanied by normal thyroid function tests in all patients. Ultrasound imaging defines a hypoechoic mass [56], which manifests as a cold nodule on scintigraphy. Per Takayama et al. [61, 63], CT scans performed in cases of leiomyosarcoma revealed a low-density tumor with calcification and necrosis infiltrating the thyroid cartilage.

Cytologic analysis of FNA biopsy specimens from malignant SMT are characterized by the presence of atypical spindle cells. These cells may be associated with increased mitotic activity and possible background necrosis. Leiomyosarcomas are typically larger and softer than leiomyomas, with increased tumor necrosis, hemorrhage, and cystic degeneration [58–60, 66]. The differential diagnosis includes anaplastic carcinoma of the thyroid. It is also important to rule out the spindle cell variant of medullary thyroid carcinoma, which is usually associated with high plasma calcitonin levels, and shows a specific immunocytochemical profile with positivity for calcitonin, CEAm, and negativity for thyroglobulin.

Histologic examination reveals fascicles of spindle-shaped smooth muscle cells, often connected to vessel walls. Tumor cells exhibit hyperchromatic, blunt nuclei centered in the cytoplasm, sometimes next to vacuoles. Leiomyosarcoma is defined by notable nuclear atypia, high mitotic rate, extensive necrosis, and vascular or capsular invasion.

Immunohistochemistry for benign and malignant SMTs includes positive reactivity to smooth muscle actin, muscle-specific actin, smooth muscle myosin, basal lamina components (including laminin and type IV collagen), and desmin, but their tumor cells are negative for cytokeratin, thyroglobulin, chromogranin, CEAm, and calcitonin [62, 67, 69].

Leiomyomas, being benign tumors, are treated with lobectomy or partial thyroidectomy alone. Leiomyosarcomas are more aggressive neoplasms often with dismal outcomes, despite drastic treatment. Thompson et al. [60], analyzing five cases of SMT including one leiomyoma, and four leiomyosarcomas, reported that their patient with a leiomyoma survived 11 years without any local or distant recurrence, while three out of the four patients with leiomyosarcoma had a survival time of less than 2 years, and the remaining one patient developed lung metastases at the 10-month follow-up.

### Peripheral nerve sheath tumors

Peripheral nerve sheath tumors (PNSTs) include benign (neurilemoma or schwannoma) and malignant (malignant peripheral nerve sheath tumor [MPNST]) neoplasms that develop from sympathetic or parasympathetic nerves [70, 71]. These tumors are mostly found in the temporal bone and upper cervical area, with approximately half appearing in the head and neck region. Perithyroid nerves can give rise to a PNST [71, 72], while primary PNST of the thyroid gland itself is exceedingly rare [73]. To the best of our knowledge, 17 patients including 16 adults and one 12-year-old child have been diagnosed with a primary PNST [74–87]. Some studies have confirmed that Schwann cells in the cervical plexus, which innervate the thyroid and sensory nerves, are the originating cells [6, 87]. Schwannoma typically presents as a slow-growing thyroid nodule that primarily affects the right lobe of the thyroid, but rare cases have been observed in the left lobe [86]. The majority of patients do not experience pressure symptoms, and a physical examination typically reveals a movable, nontender mass in the front portion of the neck. Ultrasound examination of schwannomas reveals a hypoechoic mass [88–92], while CT scans reveal a well-defined, uniform area with low density [84].

FNA is sometimes ineffective for diagnosing PNST due to the presence of limited, dispersed spindle cells in a haemorrhagic background. One example also showed obscuring inflammatory cells and contained hemosiderin, indicating an inflammatory or degenerative process [91]. Grossly, these neoplasms are gray-tan masses, often with cystic degeneration. Tumors can reach a maximum size of 7 cm. Histologically, schwannomas reveal an encapsulated neoplasm showing two patterns: Antoni A and Antoni B areas. Antoni A, the primary component in most PNST cases, is made up of intersecting fascicles of spindle cells arranged in a palisading or organoid structure (Verocay bodies). Antoni B shows tumor cells separated by edematous cystic spaces. Mitoses are typically absent, and no vascular invasion is observed.

MPNST, the malignant variant of schwannoma, is a very aggressive tumor that destroys the thyroid parenchyma and invades perithyroid tissues. MPNST can cause dyspnea due to tracheal compression and deviation, dysphagia from esophageal invasion, and dysphonia from recurrent laryngeal nerve paralysis [90]. CT scans show a non-homogeneous, low-density mass with tracheal compression and penetration of perithyroid soft tissues [87, 88, 90]. These tumors exhibit high cellularity, high mitotic activity, necrosis, and extensive vascular invasion. This type of malignancy is characterized microscopically by plump, nearly epithelioid cells surrounding capillaries, as well as necrosis with uneven boundaries and palisading at the edges. Two examples of the “Triton” type of MPNST, which is characterized by rhabdomyoblastic differentiation, were detected in the thyroid [93, 94].

Immunohistochemical analysis reveals that PNST cells are highly reactive to S- 100 protein, but not to thyroglobulin, TTF- 1, cytokeratin, chromogranin, calcitonin, actin, or desmin. Surgery is the best therapeutic option with a lobectomy in case of schwannoma, or a total thyroidectomy with extensive neck dissection in cases with MPNST. All reported thyroid schwannomas have shown a benign behaviour. Conversely, MPNST despite surgery and irradiation behave poorly, like other undifferentiated cancers [87, 88, 90, 93].

### Follicular dendritic cell tumor

Follicular dendritic cell tumor (FDCT) is a rare tumor characterized by follicular dendritic cell-like morphology and immunophenotype [90–96]. Specifically, follicular dendritic cells, found in both primary and secondary lymphoid follicles, play a crucial role in the immune response by delivering antigens to the B-cell compartment [94]. The major difficulty in diagnosing and managing FDCT is attributed mostly due to its rarity. These tumors typically originate in cervical lymph nodes, but can also affect the tonsils, pharynx, parapharyngeal soft tissue, mouth, and parotid gland [95, 96]. In 1999, Galati et al. [81] reported a case of thyroid dendritic cell sarcoma in an adult patient presenting with a painless thyroid lesion. According to the largest published series, including 34 cases of head and neck FDCT, Vargas et al. [95] found a mean age of 38 years (range, 13–73 years) and equal gender distribution.

The gross appearance of FDCTs frequently shows a tan-gray, well-defined mass. Light microscopy shows a consistent distribution of oval to spindle cells in diffuse, fascicular, and faintly whorled growth patterns. Tumor cells have eosinophilic cytoplasm, round to ovoid nuclei with localized atypia, and conspicuous nucleoli. Small lymphocytes are typically distributed between tumor cells and exhibit strong perivascular cuffing. Scattered multinucleated giant cells may be found. Foci of necrosis are uncommon. Mitotic rates range from low to high.

Immunohistochemical analysis is critical for this diagnosis showing diffuse immunoreactivity for CD21, CD23, CD35, and EMA, as well as variable positivity for S- 100 protein and CD68. They may also show focal positivity for CD45 and CD20, but not for actin, myeloperoxidase, ALK- 1, CD1a, CD3, CD5, CD30, HMB- 45, desmin, actin, thyroglobulin, or low-molecular weight cytokeratins. The intra-tumoral lymphocytic population exhibits a mix of T and B cells [90–95].

The elective treatment for FDCT is defined by surgical resection of these tumors, followed by chemotherapy and/or radiation therapy. Even though FDCT was first thought to be a low-grade malignancy, Galati et al. [97] found FDCT in four out of 17 resected cervical lymph nodes. Consequently, these tumors seem to be more likely associated with a high recurrence rate and substantial metastatic potential [98], and hence are best managed as a head and neck tumor with a moderately aggressive course of treatment [95, 96].

### PTC with spindle features

Papillary thyroid carcinoma (PTC) is the most common malignant tumor of the thyroid gland, accounting for at least 80% of cases, including classic PTC and other subtypes [99–102]. Among the histological variants identified so far that may exhibit a spindle cell morphology, a spindle cell component is seen in PTC with spindle cell metaplasia, PTC with fibromatosis/fasciitis-like stroma (NFS), cribriform morular PTC.

**Fig. 4:**
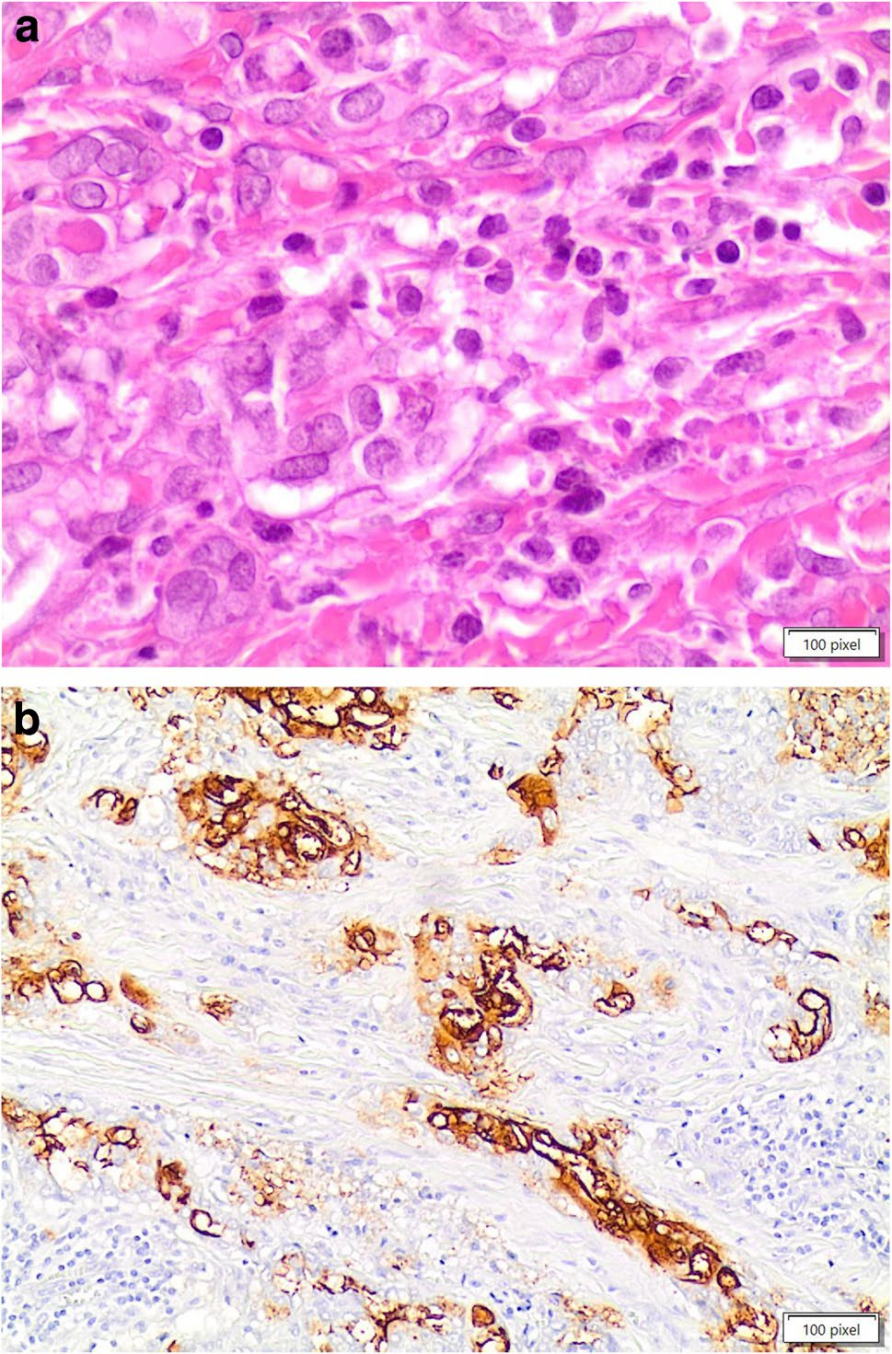
**A** and **B**: panel **a** shows a papillary thyroid carcinoma with a spindle cell component and the classic PTC nuclei (H&E 40 ×). Panel **b** shows the positivity-membranous-for HBME- 1 in the same case (**a** and **b**, 40 ×)

The association between PTC and spindle cell metaplasia is very unusual with unknown prognostic value (99–102; Fig. 4a–b). The metaplastic transformation of thyroid follicular cells toward spindle cell cytomorphology appears to be attributable to cell–matrix interactions during neoplastic growth that are under the influence of cytokines produced by follicular neoplastic cells [103]. PTC with spindle cell metaplasia is more frequent in females, with a preference for afflicting adult patients aged 25–67 years [99–101, 103]. These unique tumors range in size from 3 to 50 mm [103]. Histologically, most PTC nodules with spindle cell metaplasia show an encapsulated lesion, composed of spindle cells (from 1% to almost 100% of the entire tumor) with a multicentric, nodular, or diffuse infiltrative growth pattern intermingled with scant and isolated trapped PTC follicles and/or minimal papillary structures. Of note, these tumors only demonstrate subtle classic PTC nuclei, leading to some misdiagnoses as NIFTP or FTC. However, the evidence of minimal papillary architecture, in few cases the expansive pattern, and the expression of molecular profile of PTC, might help in the correct diagnosis. The spindle nuclei are fleshy to thin, slightly serrated, deceptively bland looking, and their nuclei have finely granular chromatin with inconspicuous nucleoli and rare pseudoinclusions; the cytoplasm of these spindle cells is broad, eosinophilic, and their cell edge is indistinct [99, 100, 103, 104].

The definitive diagnosis of this unusual PTC is made using an immunohistochemistry panel. There should be strong positivity for thyroglobulin, PAX- 8, and keratins (AE1-AE3 and CAM5.2) in the non-spindle follicular cells, while spindle cells are weakly reactive for the same antibodies. Both cellular components are strongly positive for TTF- 1, PAX 8, and bcl- 2 [99–104]. Membranous staining for E-cadherin, present in follicular cells, is lost in the spindle cell component. Spindle cells and follicular cells are negative for estrogen and progesterone receptors, desmin, CD34, CD99, CK7, calcitonin, chromogranin A, synaptophysin, S- 100, EMA, CD21, CD23, CD35, SMA, HHF35, HMB45, P63, P40, or P53. The overall Ki67 proliferation index is < 3%, showing that this PTC with spindle cell proliferation usually has the low proliferative index of the majority of classic PTC [99–104].

### PTC with fibromatosis/fasciitis-like stroma (PTC-NFS)

These PTC cases are a rare biphasic variant of PTC (0.17–0.5% of all cases), characterized by areas of conventional PTC mixed with a prominent and predominant myofibroblastic component [105–109]. The lesional spindle cells typically form a discrete mass that grows and infiltrates independently of the associated epithelial neoplasm. The spindle cells themselves are associated with stroma resembling nodular fasciitis or desmoid-type fibromatosis. The solid appearance of this spindle cell proliferation is important to differentiate from the desmoplastic stromal response that commonly occurs in PTC [106].

The first description of PTC-NFS was reported by Chan et al. in 1991; since then, approximately 30 cases have been published in the literature [106, 107]. There is a prevalence of adult patients with a higher incidence among women (F:M ratio of 3:1) and a tumor size ranging between 2 and 10 cm [106]. Macroscopically, these tumors present as a well-circumscribed, non-encapsulated tumor [108]. Histologically, PTC-NFS is characterized by a double pattern: (1) minor glandular and papillary features of classical PTC and (2) predominant reactive stromal component consisting of mild spindle cells arranged in parallel or wavy fascicles, or in bundles intertwined with collagen fibers. The spindled cells have abundant cytoplasm, oval or slender nuclei, delicate chromatin, and small clear nucleoli. Spindle cells may also show mild atypia and occasional mitoses. Additionally, a small number of peri-lesional lymphocytes may be observed [106, 107]. Based on immunohistochemical evaluation, the epithelial component shows positivity for TTF- 1, thyroglobulin, galectin- 3, HBME- 1, mutant protein BRAF V600E (strong cytoplasmic diffuse granular positivity), beta-catenin (weak membranous positivity), CK7, BCL2, AE1/AE3 (strong and diffuse membranous positivity), and PAX8 (variable nuclear staining) [106–108].

Spindle tumor cells show prominent cytoplasmic staining for smooth muscle actin (SMA), vimentin, CD34, and calponin, diffuse nuclear staining for SOX11, and significant nuclear and cytoplasmic staining for β-catenin, which globally support that the spindle cells are fibroblasts and myofibroblasts, which can produce collagen [106–108]. Spindle tumor cells are negative for BCL2, BRAF, and PAX8. Desmin, CD31, CD34, CD117, p53, and S100 are negative in both components [91–93]. Both patterns are characterized by a very low proliferation index of Ki- 67 (< 3–5% in both the epithelial and stromal components) [107–109].

Molecular analysis shows the presence of *BRAF*^*V600E*^ mutation in the epithelial component, which is supposed to play a role in the amount of associated myofibroblastic stroma [107–109]. Furthermore, the detection of CTNNB1 (β-catenin) mutations in the stromal component [107, 108] genetically subclassifies these tumors into two separate subcategories: (1) PTC-DTF, associated with CTNNB1 mutations, and (2) PTC-NFS without this genetic alteration.

Of note, the diagnosis of PTC-NFS requires a combination of ultrasound, cytology with FNA, and histological examination to distinguish it from other similar benign and malignant thyroid diseases [106, 107]. The main differential diagnosis is anaplastic thyroid carcinoma, which can be distinguished based upon its lack of stromal nuclear beta-catenin expression, lack of follicular epithelial differentiation, negativity for PAX8 and/or TTF1, and the presence of increased proliferative activity.

As for classic PTC, surgical treatment involving total thyroidectomy combined with cervical lymph node dissection is the best option for PTC-NFS [106], followed by iodine 131 therapy if there is any local or remote spread of disease. The necessary follow-up time in most cases is around 1 year. The prognosis of patients is favourable, similar to classic PTC [106].

### Cribriform-morular thyroid carcinoma (CMTC)

CMTC is a rare and unusual thyroid malignancy, with a peculiar growth pattern secondary to permanent activation of the WNT/β-catenin pathway, associated with aberrant nuclear expression of β-catenin, because of the mutation of its gene (CTNNB1) [110–114]. Distinct from previous WHO editions, where CMTC was included among the PTC variants, the new 5 th edition of the WHO classification of endocrine and neuroendocrine tumors considers CMTC to be an independent thyroid neoplasm of uncertain histogenesis. Recently, differentiation related to the thymic/ultimobranchial sac has been postulated in the histogenesis of CMTC, given the co-expression of CK5 and CD5 in the morular component, together with positivity for CDX2 (recently detected in a subset of thymic carcinomas) [111, 113, 115–117].

CMTC is also associated with familial adenomatous polyposis (FAP), but rarely can be sporadic [110–115]. About 1% of patients with FAP may have thyroid cancer, mostly CMTC, preceding the development of colonic polyposis by 4–12 years in 40% of the cases. Based on this strong association, colonoscopy and analysis of the APC gene in patients with CMTC is recommended [14–16, 110–113].

CMTC commonly occurs in young women, usually patients younger than 40 y/o (average age is 26–33 years) [110–113, 116]. Generally, the familial form of CMTC associated with FAP is manifested in younger patients and is multifocal and indolent, while the sporadic form presents as a solitary thyroid nodule and may occasionally be aggressive [110–116]. Most patients present with a gradually increasing painless neck mass or a thyroid mass discovered incidental to manual palpation or imaging [110, 116]. By ultrasound, CMTC presents as an oval, solid, well-defined nodule, that is circumscribed, heterogeneous and hypoechogenic, without a hypoechoic halo or calcifications. Few nodules can reveal cystic change or microcalcifications. The size of these lesions usually varies from 0.3 to 3 cm. Thyromegaly or extra-thyroid extension are very rare [110].

Cytological features include hypercellularity, a papillary arrangement of tall-columnar cells with a cribriform pattern, and the presence of moruliform features or clusters of cells with vortex formation. The spindled cells exhibit clear and dark nuclei with a frosted glass appearance, nucleoli, nuclear grooves, and peculiar nuclear clearing. Associated foamy histiocytes are notable, as well as abundant hemosiderin and hyaline material. There is an absence of colloid in the background. Confirmation of the diagnosis is supported by nuclear and cytoplasmic positivity for beta-catenin and biotin [110, 112, 115, 116].

**Fig. 5:**
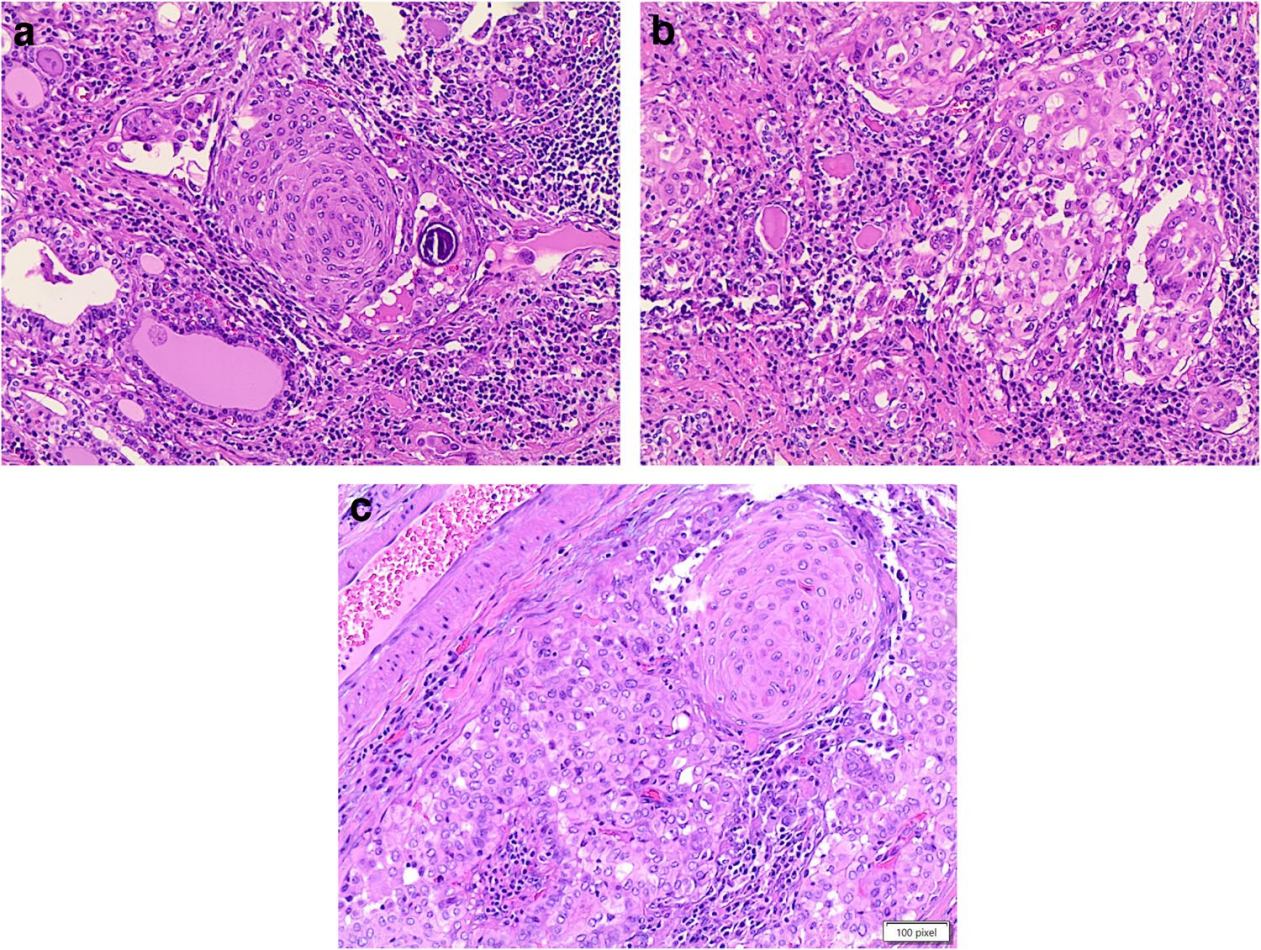
**a, b, c** The pictures show details of the malignant features of the cells and the cribriform and morular pattern of the same cells. (H&E 20 × and 40 ×)

A definitive diagnosis can be made on histopathological examination, revealing a well-circumscribed lobulated mass, with multiple satellite nodules. Generally, there is no lobe predilection [110, 116]. Microscopically, CMTC shows highly cellular areas which can range from well to poorly circumscribed with a variety of architectural patterns, (papillary, cribriform, solid, trabecular, and follicular), intermingled with scattered squamoid or morular islands (Fig. 5a–c). Some tumors show a complex fusion of different histological patterns and complex branched papillary structures coated by cuboidal or high columnar cells with nuclear pseudo-stratification and nuclear characteristics of classical PTC, which are detected even in cribriform areas (110–116; Fig. 5a–c). Histology of the cribriform areas reveals back-to-back follicles with anatomical bars and arches of anastomosed cells in the absence of intermediate fibrovascular stroma. Another common finding is represented by solid areas or morules or squamoid nests, which are typically devoid of keratinization or intercellular bridges. These tumors also lack mitotic figures, necrosis, colloid, psammoma bodies, and multinucleated giant cells. The neoplastic cells can be cuboidal or columnar, but most commonly demonstrate a spindle-fusiform shape, with eosinophilic cytoplasm. The nuclei in these cells are eccentrically located, round to oval, are optically clear, have poorly formed longitudinal nuclear grooves, small indistinct nucleoli, and typical intranuclear and eosinophilic cytoplasmic inclusions as seen in classical PTC. Tall cells and columnar cells can rarely be identified [110, 111, 113, 115].

The typical immunoprofile of CMTC shows diffuse positivity for thyroglobulin, low- and high-molecular weight cytokeratins (34βE12, CAM 5.2, CK19, and CK5/6), epithelial membrane antigen, vimentin, ER, PR, TTF- 1, β-catenin (strong nuclear and cytoplasmic positivity), and biotin [110–116]. Furthermore, immunopositivity has also been reported for neuron-specific enolases, CD15, HBME- 1, p53, galectin- 3, Bcl- 2, and Rb. On the other hand, CMTC is negative for synaptophysin, chromogranin A, carcinoembryonic antigen (CEA), and calcitonin. Morules generally do not express thyroglobulin, TTF- 1, 34βE12, PAX8, p63, p40, but express E-cadherin, p53, bcl- 2, galectin- 3, CK19 (weak), β-catenin, CK5/6, CD5, CDX2, and CD10 (weakly and focal).

Some CMVC cases have rearrangements of the proto-oncogene RET, a characteristic marker of PTC, as well as germinal mutations, somatic mutations and loss of heterozygosity of the APC gene, and CTNNB1 mutations. Interestingly, *BRAF*^*V600E*^ mutation has not been seen in CMTC. Recently, Giannelli et al. reported a mutation of K-RAS in a CMTC associated with FAP and with aggressive characteristics [94–96, 98–100], as well as PI3 K3 CA mutations [116].

Either lobectomy for the sporadic cases or total thyroidectomy for familial cases is usually the treatment of choice, without extended lymph node dissection [110, 112]. The difference in the surgical treatment is associated with evidence that sporadic CMTC has a better prognosis than other aggressive variants of PTC; although, CMTC with TERT promoter mutations do behave aggressively [111].

The 5-year and 20-year survival rates of CMTC associated with FAP are 90% and 77%, respectively. The long-term prognosis is excellent, if patients with FAP receive colonoscopy screening. The better prognosis is likely to be due to the young age of patients and the rarity of capsular and/or vascular invasion and nodal metastases [110].

### Medullary thyroid carcinoma (MTC)

MTC is a malignant tumor with neuroendocrine features, that arises from parafollicular C cells in the thyroid gland. MTC accounts for around 5–10% of thyroid neoplasms. The WHO classification of endocrine neoplasms recognizes more than 14 histological subtypes, including a spindle cell variant of MTC, characterized by very few cases and the possibility of prior misleading diagnoses [107].

In most cases (around 80%), MTC is sporadic. In the remaining 20% of cases, MTC arises in individuals with multiple endocrine neoplasia syndrome (MEN, types 2 A and 2B) characterized by the presence of a germline mutation of the RET proto-oncogene with autosomal dominant inheritance [118–129]. This mutation may also be present in sporadic MTC [120].

Clinically, MTC usually presents as one or more non-painful solid thyroid nodules, which on ultrasound appear as a hypoechogenic nodule with ill-defined margins. At the time of diagnosis, MTC is often associated with lymph node metastasis in 50% of cases and distant metastasis in 15% of cases [121–123].

**Fig. 6:**
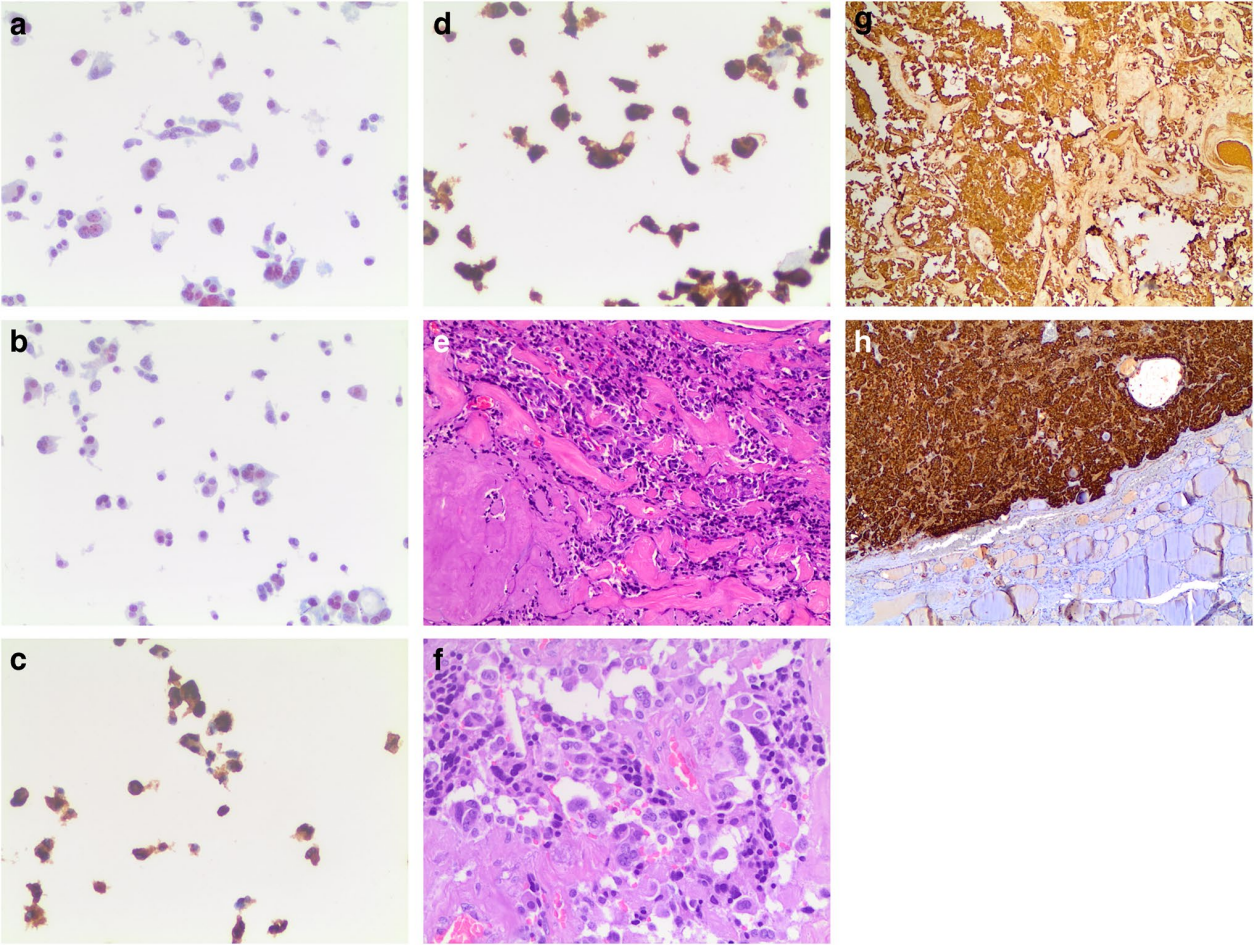
**a, b, c, d, e, f, g, h**: panels **a** and **b** showed the cytological details of a medullary thyroid carcinoma with the spindle features. Panels **c** and **d** showed the positivity for Calcitonin and CEAm on liquid based stored material (Pap stain 20 × and 40 ×, **a** and **b** 40 ×). Panels **e** and **f** showed the histological details of an MTC with the typical morphological pattern of spindle and epithelioid cells (H&E 40 ×). Panels **g** and **h** showed positivity for CEAm and calcitonin (**a** and **b** 40 ×)

Diagnostic FNA cytology samples of MTC show highly cellular smears of non-cohesive pleomorphic cells and an absence of papillary or follicular structures. Tumor cells may have a spindle, plasmacytoid, round, oval, or polygonal morphology. Their nuclei typically show scattered granular chromatin, and deposits of extracellular amyloid may be present (124–127; Fig. 6a–d). Occasionally, tumor cells may appear monomorphic, with spindle-shaped nuclei and elongated, vacuolized cytoplasm, along with nuclear budding; in such cases, the differential diagnosis may be complex, and a MTC may accordingly be misdiagnosed as sarcoma [128].

Macroscopically, MTC appears as a well-demarcated but capsule-less and grayish-white nodule. These malignancies may also be multifocal and involve both thyroid lobes, especially in the case of MEN [129, 130].

Histologically, MTC is characterized by architectural organization of tumor cells in sheets, nests, or trabeculae. These tumors consist of round or oval cells, have small nuclei with finely granular chromatin, inconsistent nucleoli, eosinophilic and granular cytoplasm, and may have scattered mitoses (Fig. 6e–h). In addition, lympho-vascular invasion is often observed. Very rarely, necrosis and hemorrhage are observed. MTC can mimic other thyroid neoplasms owing to the many variants that have been described [131, 132].

Immunohistochemical investigation should always be performed for a definitive diagnosis of MTC. MTC is positive for cytokeratins, specific nuclear enolase, synaptophysin, chromogranin, and carcinoembryonic antigen (CEA) (Fig. 6c–d, g–h). The two most sensitive immunohistochemical markers are calcitonin and calcitonin gene-related peptide; even though, they can be positive in several endocrine neoplasms of nonthyroidal origin [133–135].

In 2021, Wang et al. described four cases of a spindle cell variant of MTC characterized histologically by monomorphic spindle cells with elongated nuclei, an inconspicuous nucleolus and abundant cytoplasm, that were organized in fascicles or intertwined bundles within a fibrovascular stroma or deposits of amyloid substance. By immunohistochemistry, these spindled tumor cells were positive for calcitonin, chromogranin, synaptophysin, CD56, and TTF- 1 in all four cases described. In three of these cases, positivity for CEA was also observed, while only two cases were positive for AE1/AE3 and vimentin. Ki- 67 ranged from 3 to 10%. Three of the reported patients underwent total thyroidectomy and cervical lymphadenectomy, and one patient underwent just thyroid mass resection. At the time of study publication, all patients were still alive. The spindle cell variant of a MTC must be recognized and distinguished from other entities with spindle cell morphology that may affect the thyroid gland, such as spindle cell atypical thyroid adenoma, spindle epithelial tumor with thymus-like differentiation, poorly differentiated carcinoma, and anaplastic carcinoma, which are negative for neuroendocrine markers and calcitonin on immunohistochemistry.

### Anaplastic thyroid carcinoma (ATC)

ATC is defined by the WHO as a highly aggressive thyroid malignancy [136–138], composed of undifferentiated follicular thyroid cells [120]. ATC comprises 1.7% of all thyroid tumors [138]. ATC is the most aggressive thyroid tumor, responsible for more than half of the cases that result in annual thyroid cancer-related mortality. They are associated with a disease-specific mortality of nearly 100% [137]. Usually, ATC affects elderly people, with a mean age in their mid- 60 s [4]. There also tends to be a female predominance (female to male ratio is 1:2) [136]. Recent studies suggest there has been an increase in incidence in the last few years [139], even though no specific cause(s) for this phenomenon has been found.

Clinically, the primary symptom of ATC is a rapidly enlarging neck mass that may cause local pain and tenderness. Moreover, these aggressive tumors may be associated with compression of the upper aerodigestive tract, resulting in dyspnea, dysphagia, hoarseness, and cough with or without hemoptysis [140].

Although ATC can arise de novo, approximately 20% of afflicted patients have a history of differentiated thyroid cancer, and 20% to 30% have a coexisting differentiated thyroid tumor, which has led investigators to believe that ATC represents the final step of a multistep process of dedifferentiation, with activating driver mutations in *BRAF* and *RAS* genes in the early phases and of secondary oncogenic mutations of *TP53*, *TERT* promoter, EIF1 AX, and PIK3 A genes in later events [141, 142].

**Fig. 7:**
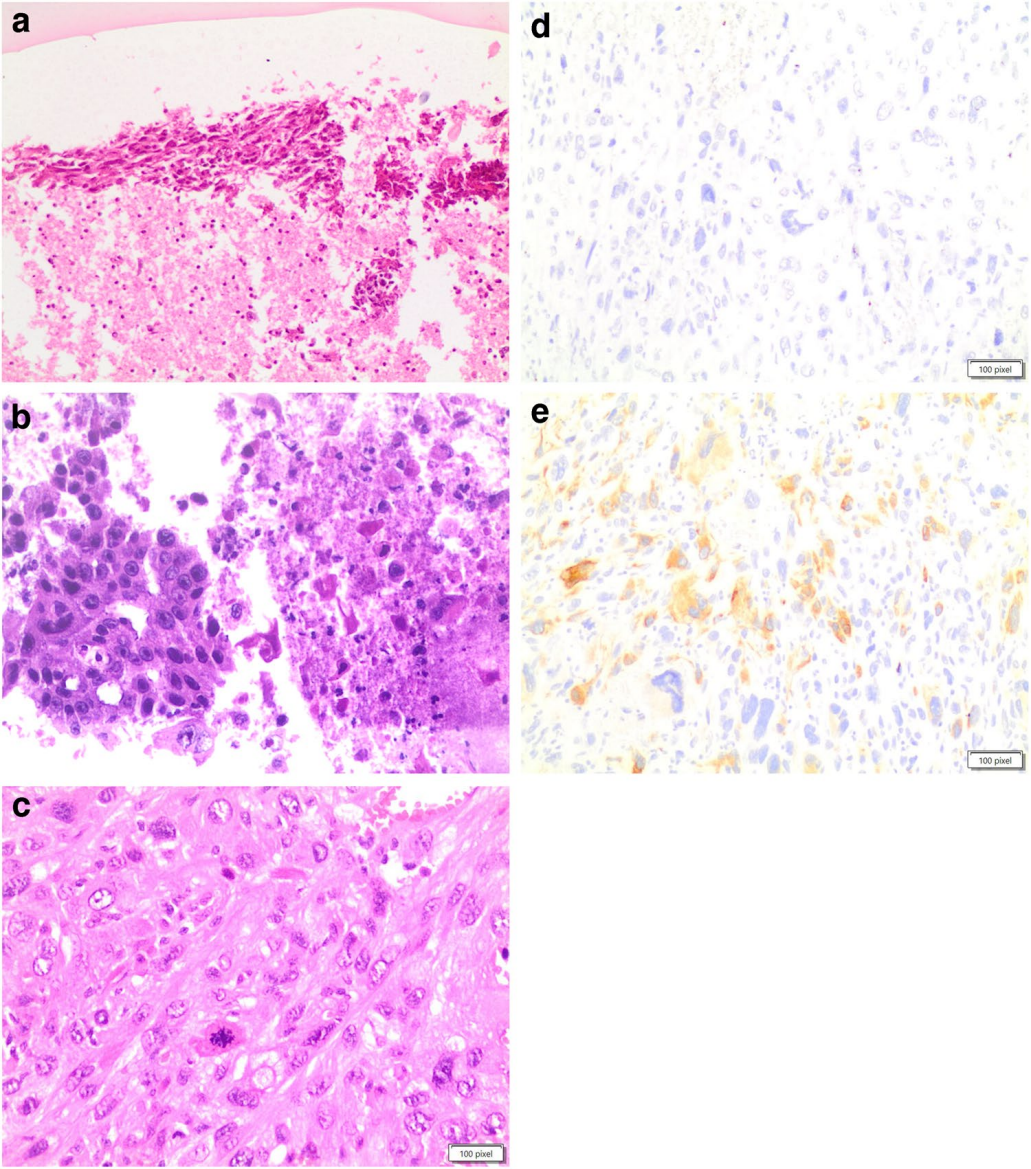
**a, b, c, d, e**: panels **a** and **b** showed the pleomorphic features of an anaplastic carcinoma on cell-block obtained fro liquid based stored material (H&E 40 ×). Panel **c** shows the morphological details on a case with histology (H&E 40 ×). Panel **d** shows negativity for thyroglobulin (**a** and **b** 40 ×), while panel **e** shows positivity for CAM 5.2 (**a** and **b** 40 ×)

ATC is a high-grade carcinoma that shows different morphological patterns including epithelioid, spindle, giant cell, pleomorphic, squamous and the rarer paucicellular, rhabdoid, angiomatoid, and metaplastic chondroid or osteoid differentiation. It is not unusual to have a mixture of spindle and giant cells together with other morphological features (139; Fig. 7a–e). All ATC cases are characterized by infiltrative growth, often with extrathyroidal extension and vascular invasion, as well as striking nuclear pleomorphism, increased mitotic activity with atypical mitoses, and extensive coagulative tumor necrosis.

**Fig. 8:**
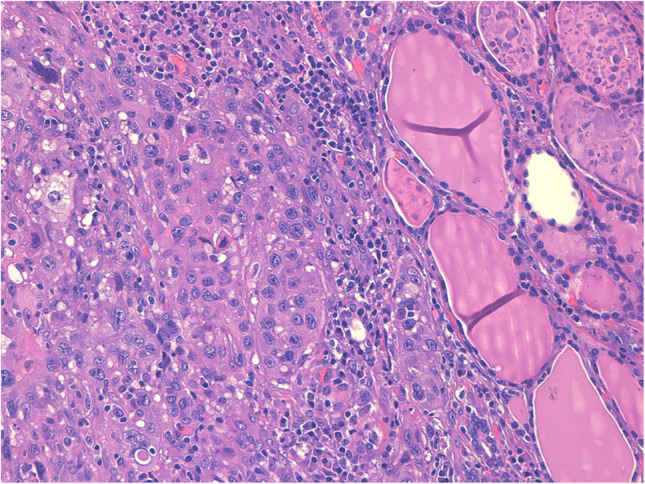
The pictures shows the details of the squamous cell variant of anaplastic thyroid carcinoma (H&E 40 ×)

Spindle cell morphology consists of a proliferation of neoplastic cells arranged in a fascicular or storiform architecture, with a rich vasculature comprised of thin delicate branching vessels, sometimes with broad spaces imparting a hemangiopericytoma-like pattern. It is worth mentioning that two other subtypes of ATC, paucicellular and squamous/squamoid, can have a spindle cell morphology [144, 145]. The paucicellular carcinoma variant is defined by substantial fibrosis and sclerosis and low cellularity composed of fusiform cells with mild atypia that bears a resemblance to fibroblasts or myofibroblasts, accompanied by scattered lymphocytes and areas of infarct-type necrosis [144, 145]. Squamous cell carcinoma of the thyroid, previously considered a separate entity by the WHO, is now regarded as a subtype of ATC, characterized by obvious keratinization and intercellular bridges (Fig. 8). This unique tumor can show spindle cell components arranged into bundles with marked cellular atypia, nuclear pleomorphism, and many mitotic figures, along with neurovascular invasion and fibrous hyperplasia [147].

Due to a lack of a pathognomonic morphology, immunochemistry (IHC) plays a fundamental role in diagnosing ATC even though morphologically, ATC is much more pleomorphic that other thyroid cancers, and it has a higher mitotic rate with more atypical mitoses than poorly differentiated thyroid carcinoma. Compared to poorly differentiated thyroid carcinoma, ATC shows negativity for thyroglobulin (Fig. 7d) and TTF1; although, some authors have reported focal and weak positivity for TTF1 in 10–30% of cases; further, the Ki67 proliferation index is generally high (more than 30%) and p53 is overexpressed in more than 50% of cases [140, 141, 147]. Most ATCs shows keratin expression, such as AE1/AE3 and CAM 5.2 (Fig. 7e) staining. Some authors have reported a higher rate of positivity for keratin cocktails, detecting low molecular weight keratins such as CAM 5.2 [140, 147]. When immunochemistry cannot demonstrate the epithelial nature of ATC, the diagnosis can be very problematic. Considering that primary sarcomas of the thyroid are very rare, it has been suggested that all sarcomatoid tumors of the thyroid gland best be considered to represent ATC until proven otherwise [143]. The squamoid variant of ATC is morphologically indistinguishable from true squamous cell carcinoma (Fig. 8). In this case, PAX8 is helpful since it is expressed in 35–80% of ATC cases [140].

*BRAF V600E* mutation, which occurs in 50% of well-differentiated papillary thyroid carcinomas, are detected in 25–30% of ATC. IHC for BRAF V600E is accordingly extremely useful in diagnostic practice, for its strong concordance with BRAF status [139, 140].

### Angiosarcoma

**Fig. 9:**
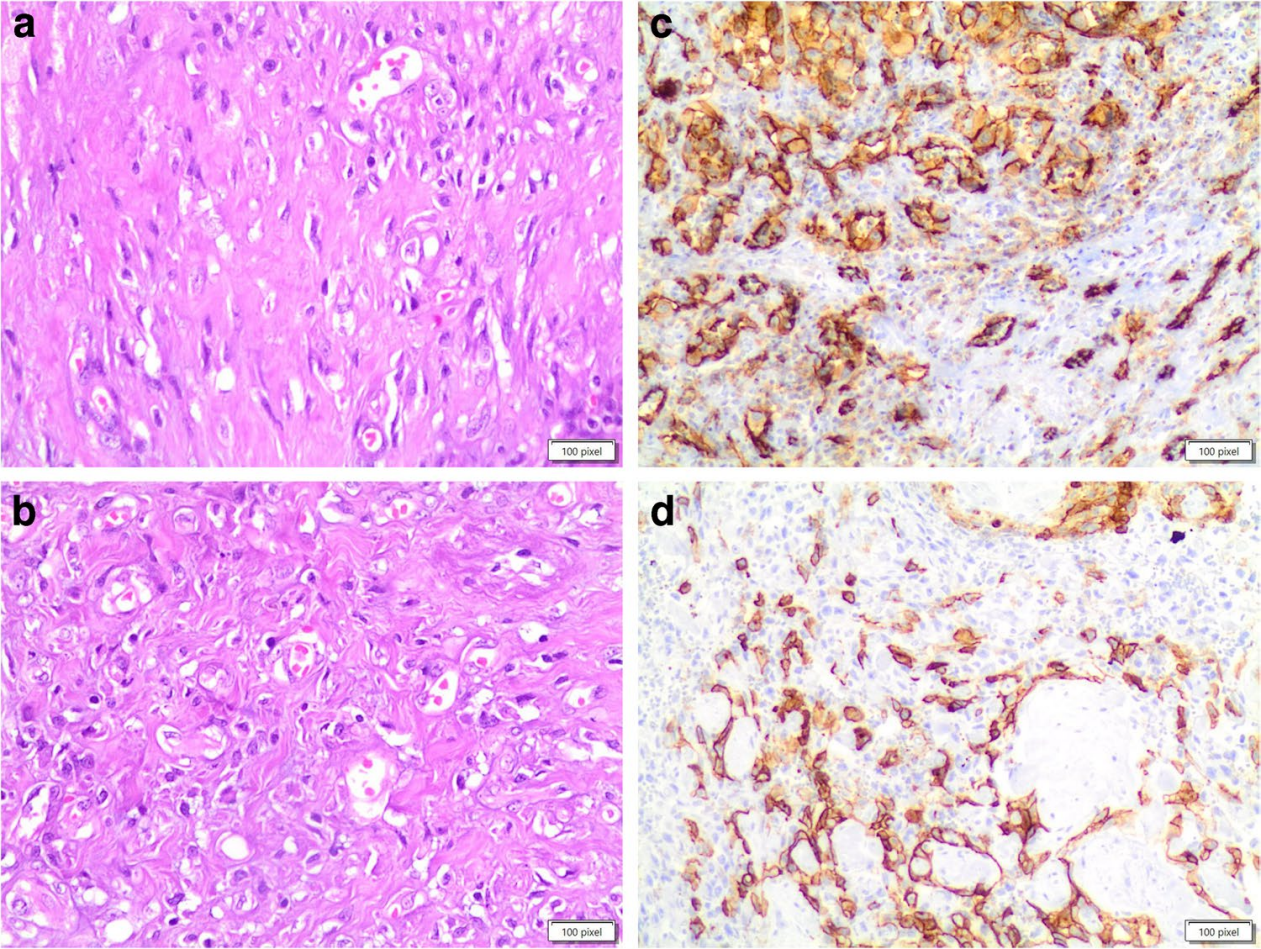
**a, b, c, d**: panels **a** nd **b** show the morphological proliferation of malignant endothelial cells composing the angiosarcoma (H&E 40 ×), while panel **c** shows positivity for CD31 and panel **d** positivity for CD34 (**a** and **b** 40 ×)

Thyroid angiosarcoma is a rare tumor that occurs largely in European Alpine regions, mostly linked with iodine deficiency, and it accounts for 10% of all thyroid neoplasms [148, 149]. Data from the literature reported that the age of afflicted patients ranges from 50 to 88 years, with a 9:3 female-to-male ratio [150]. The clinical and radiological features of these neoplasms show a solid and solitary nodule that compresses the thyroid. Histological findings demonstrate a poorly differentiated pleomorphic tumor with irregular vascular gaps and anastomosing channels lined by atypical endothelial cells (Fig. 9a–b). It is imperative in these cases to rule out ATC. Specifically, distinguishing between angiosarcoma and angiomatoid ATC is challenging because they share histological and immunohistochemical features, and also have the same prognosis. However, angiosarcoma tumor cells invariably show immunoreactivity for one or more endothelial markers (factor VIII, CD31, CD34; Fig. 9c–d), as well as epithelial markers such as cytokeratins and concomitant negativity for thyroglobulin or podoplanin (D240) [150]. Tumor cells in angiomatoid ATC display epithelial markers (cytokeratins, EMA), and occasionally stain with endothelial markers as well as PAX8, TTF1, and podoplanin [149, 151–156].

Cutlan et al. suggested proposed criteria in order to distinguish angiosarcoma from angiomatoid ATC. According to these researchers, thyroid angiosarcoma can be distinguished by the presence of endothelial differentiation and IHC positivity for vascular and epithelial markers. In contrast, angiomatoid ATC is distinguished by the expression of endothelial and epithelial markers, as well as thyroglobulin. If the tumor in question does not express any vascular markers, according to these researchers it is best classified as an ATC with an angiosarcoma-like appearance [151].

The distinction between primary and secondary angiosarcoma involving the thyroid gland is based largely on epidemiological and clinical factors. In fact, the most common site of angiosarcoma is the skin of the head and neck, accounting for around 60% of all cases, whereas thyroid angiosarcoma is extremely rare. Second, the chronology of symptoms can be useful. Thyroid angiosarcoma is an extremely aggressive tumor. Indeed, it can quickly metastasize to the cervical lymph nodes, lungs, and brain, or it can spread to the gastrointestinal tract, causing serious bleeding [154–156]. Surgical resection is often combined with adjuvant chemotherapy and/or radiotherapy as the standard of therapy.

### Fibrosarcoma

Primary thyroid fibrosarcoma is extremely rare [157, 158], with a peak in elderly patients, between 60–79 years of age [159, 160]. Nonetheless, Postovsky et al. reported a case in a 14-year-old pediatric patient with undifferentiated thyroid sarcoma [161]. Among the various thyroid sarcomas, Surov et al. reported that thyroid fibrosarcoma accounts for 9.2% of them [159]. There are no specific clinical or radiological findings to help with this diagnosis. Although soft tissue sarcomas are known to grow slowly, there have been some examples in the literature in which these tumors grew quickly, causing breathing and swallowing difficulties due to painless pressure [160, 162]. Darouassi et al. reported a case involving a rapidly developing tumor in thyroid tissue that covered the entire neck within 2 months since hospital admission [162]. General symptoms included dysphagia and tracheal compression [157, 160, 162].

Although US and CT can reveal a mass attributed to fibrosarcoma, they do not provide precise information to support a definitive diagnosis. Surov et al. found that sarcomas can be hypo- or hyperechoic with US imaging, and hypo- or hyperdense with CT scans [159]. The cytological evaluation of procured samples is characterized by neoplastic cells which might resemble mesenchymal, histiocytic, epithelial, or lymphoid in origin [157]. Immunohistochemistry should thus be used to make a definitive diagnosis of fibrosarcoma [163]. Prognosis is tightly related to tumor cell structure, cellular pleomorphism, mitotic activity, and necrosis [167]. On histology, the samples show a mixture of cells with minor atypia intermingled with atypical spindle cells with many collagen fibers, classically growing in a herring bone pattern. These spindle cells show strong vimentin positivity and are also negative for EMA, suggesting the distinction can be made between carcinoma and conventional fibrosarcoma [157, 160, 162, 163]. Furthermore, negative staining with HMB45 aids in the differential diagnosis of malignant melanoma [157]. Chemotherapy and radiotherapy have been used in a subgroup of these patients not eligible for surgery, but their effectiveness is debatable [160, 162, 164].

### Synovial sarcoma

Primary thyroid synovial sarcoma (SS) is a rare tumor. SS is notable for recurrences after a few years of treatment [165]. These tumors mostly affect people during adolescence and early adulthood, with a 2:1 M/F ratio. In past decades, especially before 1940, researchers have documented that most thyroid anaplastic carcinomas were wrongly diagnosed as SS [166]. To the best of our knowledge, only 12 cases of primary thyroid SS have been reported in the literature. The most prevalent clinical feature of SS is a rapidly developing neck tumor that resembles ATC. They are also clinically associated with excessive salivation, hoarseness, dysphagia, and dysphonia. Thyroid SS is difficult to diagnose due to its non-specific clinical appearance and rarity. Imaging techniques are unable to distinguish SS from other thyroid cancers. Pre-operative FNA may help render a diagnosis, provided there is sufficient cellular material to perform ancillary tests to confirm the diagnosis. Making the diagnosis of SS cytologically is challenging. For example, previous publications highlight three patients in which their SS of the thyroid were misdiagnosed as medullary, follicular, and PTC with FNA [166]. Histomorphologically, SS is divided into two subtypes: monophasic and biphasic, depending on the presence of mesenchymal and/or epithelial components. Biphasic SS, which consists of both spindle and epithelioid cells, is thought to be more prevalent and less difficult to diagnose, particularly in common sites [166]. The monophasic subtype, on the other hand, is primarily formed of spindle cells, therefore making the correct diagnosis extremely difficult. Other spindle cell sarcomas, such as leiomyosarcoma, rhabdomyosarcoma, fibrosarcoma, MPNST, and spindle epithelial tumor with thymus-like differentiation (SETTLE), are included in the differential diagnosis of monophasic SS [167, 168]. Immunohistochemically, SS is often positive for epithelial markers such as EMA, CK7, CK19, as well as vimentin, CD99, and bcl- 2, which helps distinguishing it from other sarcomas. However, epithelial cell markers have been reported to be expressed in ATC, MPNST, and carcinosarcoma [166, 167].

The most accurate method to make a definitive diagnosis of SS is using molecular genetic analysis to identify the SYT/SSX fusion transcript. In almost all cases of SS, the chromosomal translocation [t (X; 18) (p11.2; q11.2)] may be discovered utilizing frozen or paraffin-embedded tissues for real-time reverse transcriptase polymerase chain reaction (RT-PCR) or FISH (fluorescence in situ hybridization) [84]. SS is regarded as a very aggressive and infiltrative tumor. To note, the novel SS18-SSX fusion-specific antibody is reported to have high sensitivity and specificity for the diagnosis of synovial sarcoma [173].

Although ample surgical resection with tumor-free margins is recommended, the tumor’s size and proximity to important structures in the head and neck region is likely to limit a radical surgery [166, 170, 171]. Lymph node dissection is generally not suggested for sarcoma therapy unless nodal involvement is detected. Chemoradiotherapy is commonly advised for sarcomas due to their invasive nature and high risk of local and distant metastases. Adjuvant chemotherapy with ifosfomide may be considered, to help reduce the risk of recurrence [169, 172].

### Undifferentiated sarcoma (US)

US of the thyroid is an uncommon variant of thyroid sarcoma, with only 20 elderly patients recorded in the literature [174–182]. More than 80% of these reported cases were female patients, with a peak age of over 60 years, and who presented with a tumor larger than 4 cm in size [183]. A definitive diagnosis is critical to determine the prognosis and treatment strategy [174–176]. Unfortunately, ultrasonographic characteristics and FNA cytology are not specific. A core needle biopsy can help differentiate US from other neoplasms, especially ATC, by combining histopathological features and immunohistochemical stains [174, 175]. In fact, both entities may have spindle cells, show marked pleomorphism, and grow in a storiform pattern that distinguishes them from other non-mesenchymal thyroid tumors. Immunohistochemically, thyroid US is positive for α1-antitrypsin and CD68, while the majority of ATC maintain a minimal and weak positivity for keratins, even in single/isolated cells. Radical surgery is required, followed by either radiotherapy or chemotherapy. Although there is no established benefit, a soft tissue sarcoma treatment sometimes includes adjuvant radiotherapy and/or chemotherapy due to the poor outcomes of surgery alone. Furthermore, molecular testing showing a thyroid-tumor-specific mutation would be in favor of ATC, especially in absence of PAX8 and/or keratin.

### Carcinoma of the thyroid with Ewing family tumor elements (CEFTE)

Also known as adamantinomatous-like Ewing tumor, this unique tumor is considered to represent an invasive primary thyroid malignancy, resembling Ewing sarcoma of the soft tissue. CEFTEs are usually large masses, occurring in young patients, and generally associated to a favourable prognosis [184–195]. Morphologically, this pathological entity is characterized by small round/ovoid cells, and demonstrates strong and diffuse immunoreactivity for p63, CD99, and cytokeratins, but stains negatively for vimemtin, TTF- 1, thyroglobulin, and calcitonin. At a molecular level, EWSR1/FLI1 rearrangement is considered pathognomonic. Considering the possible association of EWSR1 rearrangements in PTC and the co-existence of areas with epithelial differentiation in the form of a PTC component in CEFTE, some authors have hypothesized that this observation represents a “trans-dedifferentiation” phenomenon in PTC [196]. The differential diagnosis includes small cell variants of MTC, poorly differentiated/undifferentiated thyroid carcinoma, Ewing sarcoma, and teratomas with a somatic malignancy (so called “thyroblastoma”). Unlike CEFTEs, malignant thyroid teratoma is a very aggressive multi-phenotypic small cell neoplasm, which expresses SALL4 and Glypican3, is negative for p63, and is frequently associated with DICER1 alterations [187, 188].

### Spindle epithelial tumor with thymus-like differentiation (SETTLE)

SETTLE represents a primary malignancy of the thyroid gland that occurs in young patients such as adolescents and children. This tumor is thought to originate from the branchial pouches or ectopic intrathyroid thymic remnants [189]. However, derivation of SETTLE from the thymus has been debated by some authors [190–193], based on [1] negative immunostaining for CD20, a B-cell marker expressed in epithelial cells in a proportion of spindle cell thymomas; [2] negative immunostaining for CD5, a leukocyte marker expressed by several thymic carcinomas; [3] the recent demonstration of *RAS* gene mutations in thyroid SETTLEs, not been reported in thymic epithelial tumors; and [4] absence in SETTLE of terminal deoxynucleotidyl transferase (Tdt)–positive immature lymphocytes, typically peculiar to thymomas.

SETTLE is a vaguely lobulated tumor, usually capsulated or partially circumscribed, with fibrous septa and generally shows a biphasic cellular composition featuring bland spindle cells that merge into epithelial/epithelioid cells, forming cleft-like spaces, tubules or glands with respiratory-like mucinous cysts, small papillae, trabeculae, squamous nests, and infrequently clear thin ribbons. Rarely, monophasic variants of SETTLE, including neoplasms exclusively composed of spindle cells or glandular structures, have been reported [194]. Vascular invasion/permeation may be present. On the other hand, mitotic figures, necrosis, and marked atypia are usually absent, with the exception of Kirby et al. who reported a SETTLE in a 29-year-old man with numerous mitotic figures and focal necrosis [195].

**Fig. 10:**
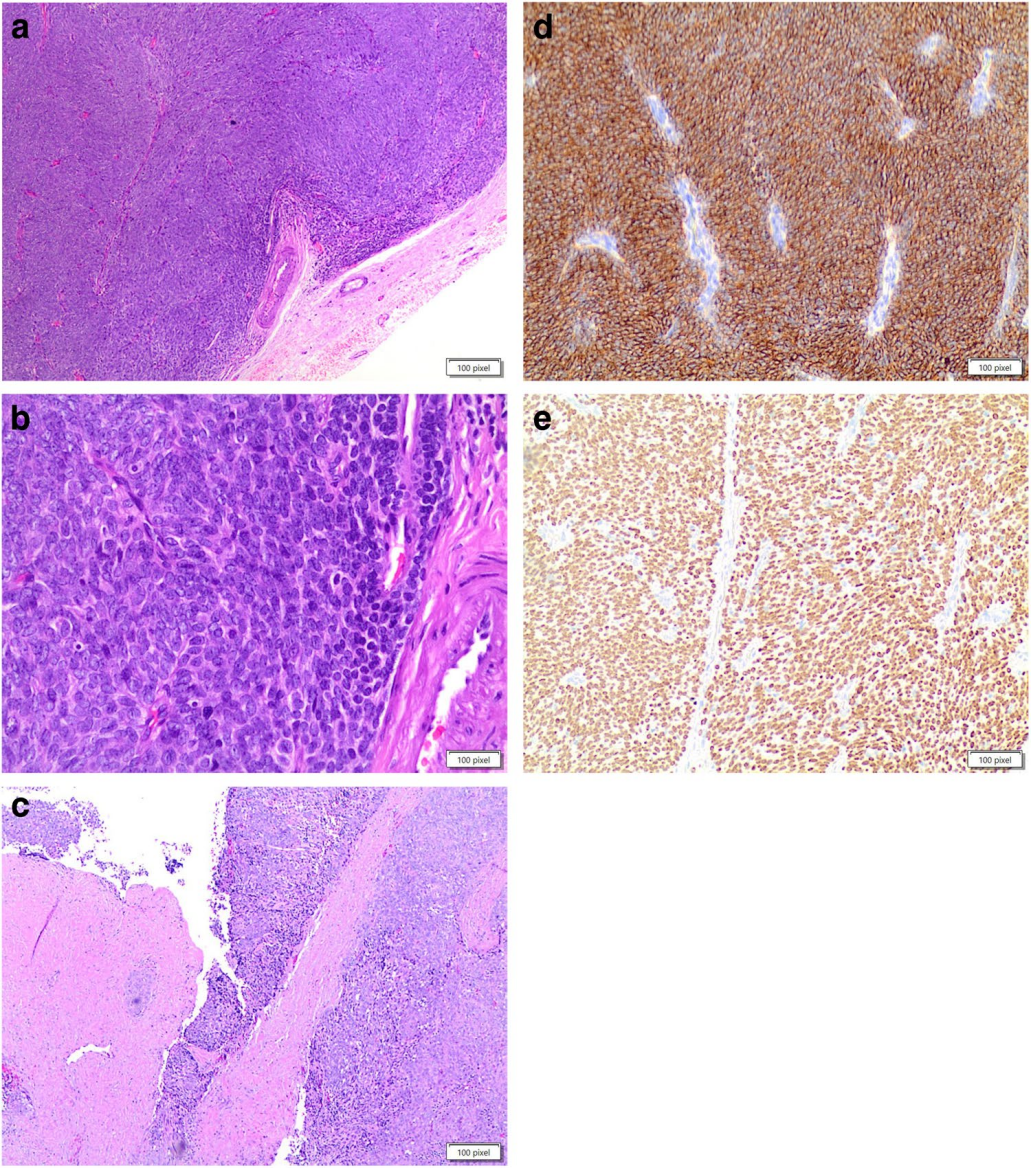
**a, b, c, d, e**: panels **a**, **b**, and **c** show the dteail of the high grade carcinoma with spindle features and thymus-like features (H&E 40 ×). Panel **d** shows positivity for keratin (**a** and **b** 40 ×), for P63 (**a** and **b** 40 ×)

By immunohistochemistry, the spindle cell component expresses low- and high-molecular-weight cytokeratins (Fig. 10d), p63 (Fig. 10e), vimentin, and CD99. Sometimes, the spindle cell component also demonstrates a diffusely positive reaction against vimentin and α-smooth muscle actin (SMA), as well as patchy positivity for muscle-specific actin (MSA), EMA, and neuron-specific enolase (NSE). Thyroglobulin, calcitonin, and TTF- 1 are usually negative. The real diagnostic challenge is the distinction between SETTLE and synovial sarcoma, which can reliably be made only after ruling out the t(X,18) rearrangement. Although SETTLE is a tumor with low-grade malignant potential, aggressive behavior has been reported in some cases, with lymph node and pulmonary metastases [196, 197].

## Conclusions

The detection of a spindle cell neoplasm or component in a thyroid lesion raises a broad differential diagnosis including many benign and malignant entities. The presence of spindle cells in a thyroid sample might represent a common or uncommon lesion. The pathology workup of such cases requires a combination of morphology (pre-operative cytopathology and/or post-operative histopathology) with the patient’s clinical history and radiological findings, together with performing ancillary techniques (e.g., immunohistochemistry, molecular testing) in order to reach the correct diagnosis and thereby appropriately manage afflicted patients.

## Data Availability

There is a word file including the details of our cases.

## Declarations

Ethical approval from the internal Committe Policlinico Gemelli 2023 n* 123.
